# Structural Changes in the Acceptor Site of Photosystem II upon Ca^2+^/Sr^2+^ Exchange in the Mn_4_CaO_5_ Cluster Site and the Possible Long-Range Interactions

**DOI:** 10.3390/biom9080371

**Published:** 2019-08-14

**Authors:** Faisal Hammad Mekky Koua

**Affiliations:** 1 Center for Free Electron Laser Science, Deutsches Elektronen-Synchrotron (DESY), Notkestrasse 85, Hamburg 22607, Germany; 2 National University Biomedical Research Institute, National University-Sudan, Air St. P.O. Box 3783, Khartoum 11111, Sudan

**Keywords:** photosystem II, redox potential, electron transfer, charge separation, photoinhibition, water oxidation

## Abstract

The Mn_4_CaO_5_ cluster site in the oxygen-evolving complex (OEC) of photosystem II (PSII) undergoes structural perturbations, such as those induced by Ca^2+^/Sr^2+^ exchanges or Ca/Mn removal. These changes have been known to induce long-range positive shifts (between +30 and +150 mV) in the redox potential of the primary quinone electron acceptor plastoquinone A (Q_A_), which is located 40 Å from the OEC. To further investigate these effects, we reanalyzed the crystal structure of Sr-PSII resolved at 2.1 Å and compared it with the native Ca-PSII resolved at 1.9 Å. Here, we focus on the acceptor site and report the possible long-range interactions between the donor, Mn_4_Ca(Sr)O_5_ cluster, and acceptor sites.

## 1. Introduction

Photosystem II (PSII) is a large multisubunit membrane protein complex with at least 19 subunits. In physiological conditions, it exists as a dimer with a molecular weight of ca. 0.70 MDa and is embedded in the thylakoid membranes of oxygenic photosynthetic organisms [1]. PSII catalyzes one of the most fundamental reactions on Earth, that is, light-induced charge separation and water oxidation [1,2,3]. PSII initiates photosynthetic reactions by absorbing light through its internal antenna pigments. Light absorption leads to charge separation of the reaction center chlorophyll pigments, the so-called P680 molecules, and the process leads to the release of an electron and the formation of P680^•+^ and Pheo^•−^ ionic radicals [4,5,6]. Then, the released electron serves to reduce the plastoquinone molecule in the Q_A_ site, and the Q_A_/Q_A_^•−^ redox couple reduces a mobile Q_B_ with two consecutive electrons in the Q_B_ site, leading to the formation of plastoquinol (Q_B_H_2_) after taking up two protons from the stroma. Q_B_H_2_ then detaches from the Q_B_ site and moves into the plastoquinol pool in the membrane-spanning region [6]. The highly reactive P680^•+^/Pheo^•−^ and the subsequent secondary radical pairs formed by charge separation can be stabilized by oxidizing the catalytic center—the Mn_4_CaO_5_ cluster, which is also called the donor site—via a redox-active tyrosine (D1-Tyr161). P680 has the highest redox potential known in biology, *E*_m_ = 1.3 V (vs. standard hydrogen electrode [SHE]), which enables it to abstract electrons from and fully oxidize two water molecules by consecutively accumulating oxidizing equivalents in the Mn_4_CaO_5_ cluster during S*n*-state transitions, where *n* = 0–4 [5,6].

**Figure 1:**
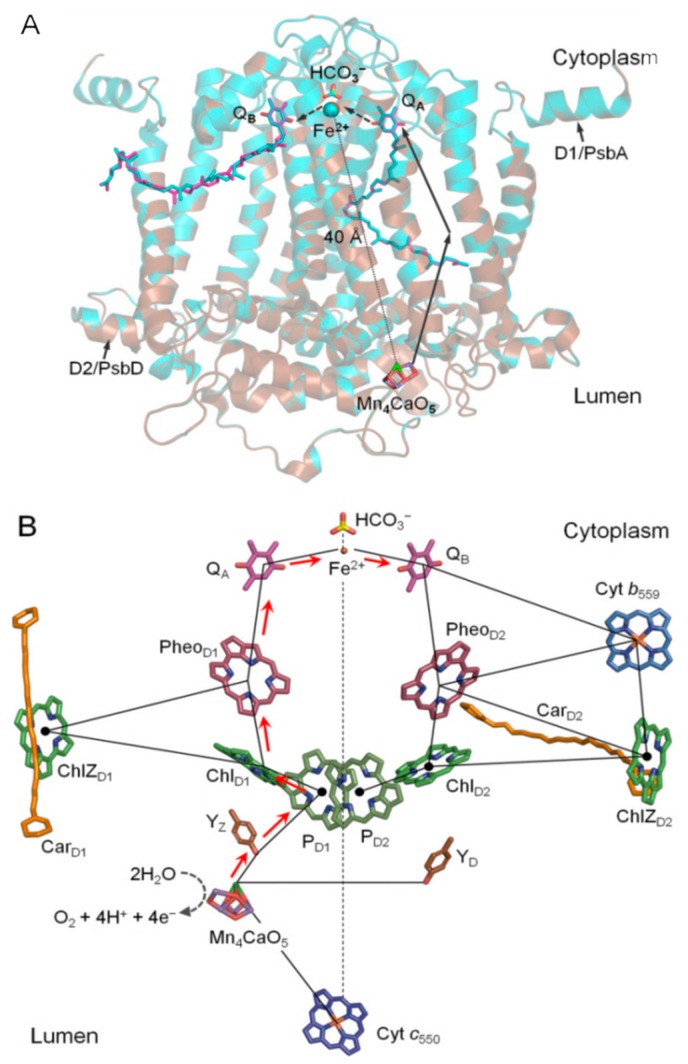
Electron transport pathway in the photosystem II complex. (**A**) Superimposed structures of the D1/D2 proteins from the native (cyan; PDB: 3WU2) and Sr-modified oxygen-evolving complex (OEC) (brown; PDB: 4IL6) photosystem II. The figure highlights the donor (Mn_4_CaO_5_ cluster) and the acceptor (Q_A_-Fe^2+^/HCO_3_^−^-Q_B_) sites, which are about 40 Å apart. (**B**) The electron transfer (ET) pathway and the locations of the main cofactors involved in the electron transfer process.

High-resolution XRD structures of PSII provide a detailed picture of the architecture of the protein matrix and the cofactor arrangements involved in the light-induced plastoquinone reduction and water oxidation processes (Figure 1) [2,3]. However, the mechanistic basis of these processes, such as the role of Ca^2+^ in PSII light-induced water oxidation, is still not fully understood. Calcium has long been known as an essential element for water oxidation [3,4,5,6,7]. For example, Ca^2+^ removal was found to suppress the formation of higher oxidation states beyond the S_2_ state [7]. This effect can be restored completely by Ca^2+^ or partially (ca. 40%–50%) by reconstituting Sr^2+^ in Ca^2+^-depleted PSII [3,7,8,9]. This implies that there is a supervening rearrangement at the donor site of PSII and likely in other locations within the electron transfer (ET) chain. Indeed, fluorescence imaging of Sr-modified oxygen-evolving complex (OEC) PSII crystals showed altered properties of functional PSII, i.e., slowdown of the ET from Q_A_ to Q_B_ and stabilized S_2_Q_A_^−^ charge recombination [10]. Consistent with this, modified ET kinetics at the donor site accompanied by conformational changes at the acceptor site were observed upon perturbations in the donor sites by Ca^2+^/Sr^2+^ (but not by Cl^−^/Br^−^) exchanges, and this exchange upshifted the redox potential (*E*_m_) of Q_A_^−^/Q_A_ by ~+30 mV [6,11]. Moreover, an even larger upshift in the *E*_m_ (Q_A_/Q_A_^−^) of ~+150 mV and a relatively smaller shift (*E*_m_ = +20 mV) in non-heme iron (NHI) were previously reported for Mn-depleted PSII, and these shifts likely affect Ca^2+^ binding to the OEC [4]. These results indicate that the ET events between the donor and acceptor sites are highly tuned and governed not only by redox agents in the ET pathway but also by the protein moiety as well as the conformational changes near the OEC. The mechanism underlying the effects of these perturbations in the Mn_4_CaO_5_ cluster on the electrochemical properties of Q_A_-Fe^2+^-Q_B_ and the structural changes that take place in the acceptor site in response to such perturbations remain largely unclear. Note that the Mn_4_CaO_5_ cluster is far (~40 Å) from the acceptor site (Figure 1) [2,3].

In our previous report on the Sr^2+^-PSII structure resolved at 2.1 Å, we focused on the conformational changes that occur in the Mn_4_Ca(Sr)O_5_ cluster and its local environment [3]. To further investigate the possible effects of Ca^2+^/Sr^2+^ exchange on the long-range interactions with the acceptor site, we revisited the structure of Sr-PSII (PDB: 4IL6) [3] and compared it with the native Ca-PSII model at a resolution of 1.9Å (PDB: 3WU2) [2]. The main focus was on the primary quinone electron acceptor (Q_A_) site as well as the local environment of the acceptor site Q_A_-Fe^2+^-Q_B_ (Figure 1). It has been reported previously that OEC perturbations due to Ca^2+^/Sr^2+^ exchange induce slight differences that are localized mainly in the OEC and nearby H-bonding networks mediated by water molecules. A significant change was observed at the W3 position: a Ca^2+^/Sr^2+^-bound water molecule, which might be responsible for the decrease in O_2_ evolution by ~60% compared with the native PSII [2,3,12]. This is reasonable because the incorporation of Sr^2+^ takes place in the OEC site, and such conformational changes are expected to reduce the activity [3].

## 2. Materials and Methods

The crystal structure of Sr-PSII was resolved at 2.1 Å as described previously [3]. The structure factor files of Sr-PSII and the native Ca-PSII structure (PDB: 3WU2) were used to compare the structures and calculate the local root mean square deviation (rmsd) of the subunits PsbA/a and PsbD/d in the two structures using the DALI server. The PsbA/a and PsbD/d subunits provide the binding sites for Q_B_ and Q_A_, respectively, as well as the NHI binding site. The overall rmsd between Sr-PSII (PDB: 4IL6) and Ca-PSII (3WU2) was calculated according to Cruickshank [13] using the SFCHECK program of the CCP4 suite [14], as mentioned in the original article [3]. In brief, the component precision index (DPI) was determined using the SFCHECK program, and the standard uncertainty value of the bond length between the two structures (rmsd) was obtained by multiplying the DPI value by the square root of 2. Further analyses and comparisons were performed using the Coot software, and the temperature factors in the selected regions of the proteins were estimated. All bond distances were obtained from the Coot program [15]. The least squares method was applied in Coot (LSQ Superpose) with the ranges of all atoms selected for the native Ca-PSII structure as a reference for comparison with the Sr-PSII mtz file. All figures were prepared using the software PyMOL (DeLano Scientific, San Carlos, CA, USA).

## 3. Results and Discussion

**Figure 2:**
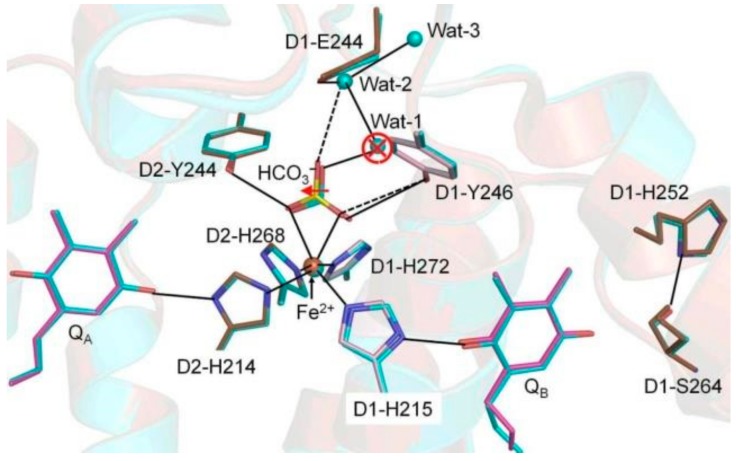
Superimposition of native Ca-PSII (PDB: 3WU2) and Sr-PSII (PDB: 4IL6) in the acceptor site pocket. The solid and dashed lines indicate that the hydrogen-bonding network within the acceptor site possibly facilitates the ET/proton transfer (PT) pathways that include two Q_A_, Q_B_, bicarbonate, non-heme iron (NHI), and water molecules. The red arrow and open circle indicate the major differences between native Ca-PSII and Sr-PSII.

**Figure 3:**
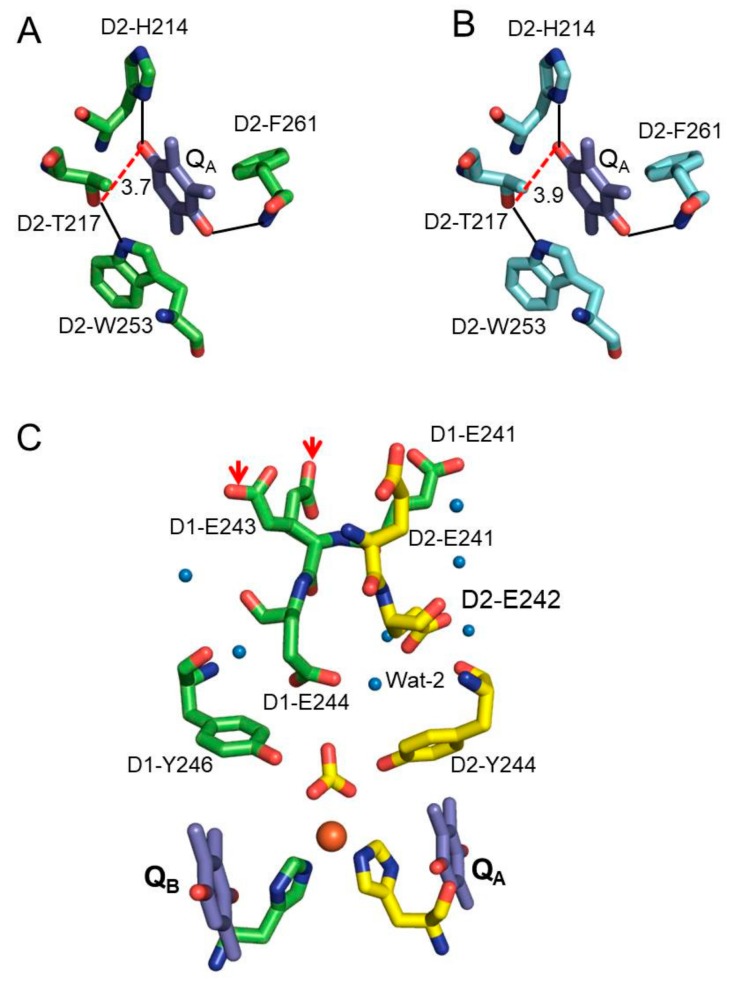
The immediate environment of Q_A_ and its relationships with the distant stromal Glu residues. (**A**) The interactions of Q_A_ with nearby residues and the additional H-bond (red dashed line) with D2-T217 and (**B**) the corresponding environment of Q_A_ in the Ca-PSII model. (**C**) The distant stromal Glu residues and associated water molecules. The red arrows indicate the rotamers of the D1-E243 residue in the Sr-PSII model.

Figure 2 shows the structural comparison between Ca-PSII and Sr-PSII in the local vicinity of bicarbonate (BCT)-Fe^2+^-His(4) and near the Q_A_ and Q_B_ sites. Q_A_ is located between the primary electron acceptor and the NHI site and mediates ET in PSII reactions (Figure 1) [2]. It is thus reasonable to attribute the shifts in the redox potential of Q_A_ to the structural changes that occur upon Mn/Ca depletion or Ca^2+^/Sr^2+^ exchanges [5,10,11,16]. Q_A_ is stabilized by van der Waals interactions, including two moderate H-bonds between its carbonyl oxygen (C=O1, proximal; C=O2, distal) and N-Phe261-D2 and N_δ_-His214-D2, respectively, as well as a π-stacking interaction with the nearby highly conserved D2-Trp253 (Table 1; Figure 3) [2,17]. This interaction is similar to the corresponding native Q_A_ interaction with slightly higher thermal motion, i.e., a 10%–20% increase in the temperature (B) factors (Table 1). This similarity indicated that the midpoint redox potential shifts (~+30 mV) that were observed upon Ca^2+^/Sr^2+^ exchange were likely due to indirect effects or other H-bonding mediators or electrostatic forces. A recent study that applied attenuated total reflection (ATR)-FTIR difference spectroscopy excluded the direct influence of Mn_4_CaO_5_ cluster perturbations on the Q_A_ site [5]. D2-Thr217 was previously predicted to form an additional H-bond with the C=O (proximal) of Q_A_ and thus contribute to the positive shifts observed in the *E*_m_ (Q_A_^−^/Q_A_) [12]. We observed that this residue formed a moderate H-bond with the indole nitrogen (N_Ɛ_1) of the nearby D2-Trp253 (~2.8 Å), whereas it interacted weakly (through weak electrostatic interactions) with the proximal C=O of Q_A_ with a shorter H-bond (~–0.2 Å) in the Sr-PSII model. This finding implied the formation of a H-bond in Sr-PSII but not in Ca-PSII (~3.9 Å) (Figure 3A,B). These differences were similar between the two monomers of each model, although the differences lay roughly within the overall rmsd value (0.2 Å) of the Cα atoms of Sr-PSII and Ca-PSII [2,3]. However, it is worth noting that the local rmsd values for the PsbD/A and Psba/d subunits between the two structures were 0.1 and 0.2 Å, respectively, as estimated by the DALI server. Such differences may give rise to different electrostatic energies between mediators and thus shift their redox potentials [18,19]. Notably, in the Ca^2+^/Sr^2+^ exchange, the *E*_m_ (Q_A_) shift was only ~+30 mV compared with +150 mV upon the depletion of Ca^2+^ or Mn(s) [4,5]. By comparing the H-bonding in the native structure with that in the Mn-depleted structure (PDB: 5MX2), we observed an even shorter bond distance (3.48 Å) between the proximal C=O with D2-Thr217, indicating the formation of an additional H-bond upon Mn depletion [20]. This agrees with the theoretical prediction and might be a cause of the upshifts in the *E*_m_ of Q_A_ [16]. Similar behavior was also observed in the H-bonding environment of the primary quinone acceptor of the bacterial reaction center (bRC) [21,22,23]. D2-Thr217 and D2-Trp253 are highly conserved between the bRC and PSII; thus, a similar mechanism is highly expected [2,24]. Interestingly, the structural changes upon Ca^2+^/Sr^2+^ exchanges or Mn depletion gave rise to weaker H-bonds between the proximal C=O of Q_A_ and the imidazole nitrogen (N_δ_) of D2-His214, with average distances of 2.79(+0.13) and 2.95(+0.29) Å, respectively [2,3,20]. The additional D2-Thr217 H-bond with Q_A_ resulted in a similar H-bonding environment between Q_A_ and Q_B_ (Figure 3A,B), which might be a cause of the decrease in the redox potential gap (∆*E*_m_) and hence a cause of the impairment of the forward ET combined with the enhancement of a direct charge recombination with P680 [25]. The perturbations of the His–Q_A_ interaction may affect their electrostatic coupling and perturb the *E*_m_ of Q_A_. Moreover, the increased stabilization of Q_A_ due to an additional H-bond from D2-Thr217 could lead to the observed positive shift in the redox potential [16]. It should be noted that such positive shifts might vary, depending on the H-bond strength and hence the stability of Q_A_. Therefore, the weak H-bond between D2-Thr217 and the C=O1 of Q_A_ in the Sr-PSII model might be the reason for the relatively smaller shifts, which were 70% lower than the *E*_m_ upshifts upon Ca/Mn removal [5,6].

We next analyzed the structural differences between Sr-PSII and Ca-PSII at the NHI site (Figure 2). NHI doesn’t participate in the ET process between Q_A_ and Q_B_. It binds four histidine residues, of which two residues—D2-His214 and D1-His215—form direct H-bonds with the proximal C=O of Q_A_ and Q_B_, respectively [2]. Several differences between the two models were observed in the NHI site; for example, significant displacement was observed in the NHI of monomer A (0.42 Å). BCT, which binds NHI with a bidentate ligand via two C=O groups, was also significantly displaced in the Sr-PSII model by an average of 0.37 Å in the two monomers (Figure 2). This displacement led to significant changes in the H-bonding network within the immediate environment of NHI. In the Ca-PSII model, BCT forms H-bonds with the phenol hydroxyls of D1-Tyr246 and D2-Tyr244 with bond distances of 3.33 and 2.99 Å, respectively. These H-bond distances were significantly elongated in the Sr-PSII model, especially the H-bond with D1-Tyr246, which was elongated by +0.31 Å (3.64 Å). The role of BCT in PSII is controversial [26], but a recent report has claimed that it is involved in the redox tuning of PSII, perhaps by modulating its binding strength during the ET [27]. This might explain the perturbed interactions between BCT and NHI (Table 1) and D1-Tyr246 upon Ca^2+^/Sr^2+^ exchange, and it is likely the reason for the positive shift in the *E*_m_ of Q_A_.

Recently, using FTIR difference spectroscopy, Kato et al. (2016) reported that the positive shifts in the mid-point redox potential of Q_A_^−^/Q_A_ might be due to modulation of the p*K*a of distant carboxylate residues in the stromal site of PSII [5]. There are indeed five Glu residues, namely, D1-Glu242, D1-Glu243, D1-Glu244, D2-Glu241, and D2-Glu242, ~15 Å from the NHI center and 55 Å from the Mn_4_CaO_5_ cluster toward the stromal side (Figure 3A,B) [2,3]. Together, these residues form extensive H-bonding networks involving several water molecules from the stromal site to the NHI center (Figure 3C). Two significant structural changes between Sr-PSII and Ca-PSII existed in this region. First, in the Sr-PSII model, the H-bonds between the C=O of BCT and the two water molecules, denoted wat-1 and wat-2, were broken as a result of the loss of wat-1 (Figure 2). Wat-1, in the Ca-PSII model, functions as a bridge between BCT and these Glu residues through an extensive H-bonding network, beginning with wat-2 and D1-Glu244 and proceeding to D1-Glu242 at the stromal site. In the Sr-PSII model, D1-Glu244 formed a weak H-bond with one C=O of BCT in the absence of wat-1 (Figure 2). Wat-1 also mediated the interaction between BCT and D1-Ser268 residue, which bind D1-His272, a ligand of NHI [2,3]. However, it was not clear whether the absence of this water molecule could cause such a shift in the redox potential of the Q_A_ site. Interestingly, wat-1 was preserved in the Mn-depleted structure of a single monomer, whereas both water molecules were absent from the second monomer [20]. The second difference was observed in one of the Sr-PSII monomers, in which the side chain of the D1-Glu243 residue formed two prominent rotamers with 0.5 occupancy for each monomer (Figure 3C). Such rotameric conformational changes may indicate the dynamic nature of D1-Glu243 and hence its involvement in the electrostatic interaction with the acceptor site. It is important to note that the similarity between the two monomers of PSII is still debated, so such differences between the two monomers cannot be excluded [28]. Further theoretical studies are required to clarify the effects of the modulation of the electrostatic interactions of these Glu residues on the redox potential of Q_A_ and their long-range interactions with the Mn_4_CaO_5_ cluster.

## 4. Conclusions

In summary, the present work highlights the structural changes that take place in the acceptor site of PSII upon perturbations of the Mn_4_CaO_5_ cluster due to Ca^2+^/Sr^2+^ exchanges. We have shown that several structural changes occur at three different levels upon Ca^2+^/Sr^2+^ exchanges: (i) the additional H-bonding at the Q_A_ site formed by D2-Thr217, (ii) the perturbations of BCT interactions with NHI and nearby Tyr residues, and (iii) the differences in the H-bonding network formed by distal Glu residues at the stromal side and the NHI binding site. Some of these structural changes might be responsible for the positive shifts in the mid-point redox potential of the primary quinone electron acceptor Q_A_ and hence impair the forward ET and enhance the backward ET and direct charge recombination between Q_A_ and P680^+^, which is important for preventing the photoinhibition of PSII.

## Figures and Tables

**Table 1:** The average interatomic distances of the acceptor site of Sr-PSII (PDB: 4IL6) and native Ca-PSII (PDB: 3Wu2) and their corresponding temperature B-factors.

Ligand	Subunit	Sr-PSII (Å)	Ca-PSII (Å)	B-factor	Sr-PSII (Å^2^)	Ca-PSII (Å^2^)
**Fe(II)**						
NE2-His215 NE2-His214 NE2-His268 NE2-His272 O1-BCT O2-BCT	psbA/D1 psbD/D2 psbD/D2 psbA/D1 psbD/D2 psbD/D2	2.06(0.02) 2.10(0.01) 2.20(0.02) 2.26(0.04) 2.30(0.02) 2.39(0.02)	2.16(0.03) 2.17(0.07) 2.28(0.02) 2.26(0.01) 2.33(0.00) 2.29(0.05)	Fe(II) NE2-His215 NE2-His214 NE2-His268 NE2-His272 O1-BCT O2-BCT	29.61(0.42) 28.48(0.17) 27.24(1.97) 27.22(1.31) 29.67(0.94) 39.41(0.77) 39.74(0.68)	27.41(0.52) 25.75(0.79) 23.72(0.20) 24.37(0.30) 28.25(0.26) 31.23(0.24) 34.04(0.23)
**Quinone B**						
O1/ND1-His215 O2/OG-Ser264 O2/N-Phe265 O2/O2-Phe265	psbA/D1 psbA/D1 psbA/D1 psbA/D1	2.50(0.00) 2.76(0.06) 2.82(0.09) 3.15(0.19)	2.48(0.06) 2.74(0.02) 2.95(0.05) 3.09(0.08)	O1/QB O2/QB OG-Ser264 N-Phe265 O2-Phe265 ND1-His215	52.37(0.55) 52.73(0.53) 48.57(0.46) 47.07(0.51) 49.90(0.49) 30.16(1.94)	60.45(0.20) 74.23(0.19) 63.62(0.17) 57.01(0.19) 66.33(0.18) 27.23(0.16)
**Quinone A**						
O2/ND1-His214 O1/N-Phe261	psbD/D2 psbD/D2	2.79(0.00) 3.02(0.00)	2.66(0.06) 2.95(0.03)	O2/QA O1/QA ND1-His214 N-Phe261	28.47(0.58) 28.60(0.62) 27.32(0.47) 26.74(0.45)	25.13(0.21) 25.60(0.22) 22.80(0.44) 23.36(0.17)

**Note:** The values presented here are averages of two monomers, and the data in parentheses are the standard deviations between two monomers.
